# Laboratory biomarkers or imaging in the diagnostics of rheumatoid arthritis?

**DOI:** 10.1186/1741-7015-12-49

**Published:** 2014-03-18

**Authors:** Ladislav Šenolt, Walter Grassi, Peter Szodoray

**Affiliations:** 1 Institute of Rheumatology, Prague, Czech Republic; 2 First Faculty of Medicine, Charles University in Prague, Prague, Czech Republic; 3 Clinica Reumatologica, Scuola di Specializzazione in Reumatologia, Ancona, Italy; 4 Università Politecnica delle Marche, Ancona, Italy; 5 Institute of Immunology, Rikshospitalet, Oslo University Hospital, Oslo N-0027, Norway

**Keywords:** Rheumatoid arthritis, Autoimmunity, Disease activity, Laboratory biomarkers, Ultrasound imaging

## Abstract

Rheumatoid arthritis (RA) is a common autoimmune disease in which a heterogeneous course and different pathogenic mechanisms are implicated in chronic inflammation and joint destruction. Despite the diagnostic contribution of anti-citrullinated protein/peptide antibodies (ACPAs) and rheumatoid factors, about one-third of RA patients remain seronegative. ACPAs belong to a heterogeneous family of autoantibodies targeting citrullinated proteins, including myelin-basic protein, several histone proteins, filaggrin and fibrin, fibrinogen or vimentin. In addition to ACPAs, antibodies directed against other post-translationally modified-carbamylated proteins (anti-CarP) were detected in up to 30% of ACPA-negative patients. Using phage display technology, further autoantibodies were recently discovered as candidate biomarkers for seronegative RA patients. Furthermore, in clinical practice, ultrasound may reveal subclinical synovitis and radiographically undetected bone erosions. To improve diagnostic certainty in undifferentiated arthritis and seronegative patients, ultrasound imaging and several new biomarkers may help to identify at risk patients and those with early disease. In this commentary we summarize recent advances in joint ultrasound and future potential of serological biomarkers to improve diagnosis of RA.

## Background

Rheumatoid arthritis (RA) is a chronic autoimmune disease characterized by persistent inflammation and joint damage with a heterogeneous course and different pathogenic mechanisms leading to common signs and symptoms [1]. In routine clinical practices, early diagnosis and recognition of inflammatory arthritis of short duration that develops to established RA in the future is sometimes difficult. In contrast to a few patients with inflammatory arthritis who may undergo spontaneous remission and some who may have a mild disease course with slow progression, more patients have moderate to high disease activity and some develop aggressive joint damage and systemic complications. Therefore, laboratory biomarkers and/or imaging assessments that would be more effective in the diagnosis of early disease are needed. Although RA is a clinical diagnosis and has no specific pathognomonic test defined so far, serological tests represent the most important parameters for diagnosis and for identification of at risk patients. Anti-citrullinated protein/peptide antibodies (ACPAs), especially in high levels, are associated with aggressive disease and together with acute phase reactants were implemented in the 2010 American College of Rheumatology/European League Against Rheumatism (ACR/EULAR) classification criteria of RA [2]. Fulfillment of these criteria thus persuades clinicians to initiate appropriate therapy early to avoid irreversible damage. Despite the high diagnostic value of ACPAs and rheumatoid factors (RFs), there is still a need for novel biomarkers to further improve the diagnosis of RA. Several novel autoantigens and antibodies that may improve early diagnosis and predict further development of the disease have been recently identified [3]. Besides clinical signs and serological tests, imaging techniques, particularly ultrasound, may improve early diagnosis of RA, particularly in seronegative patients.

In this commentary, we will attempt to summarize the role of ultrasound and several serological biomarkers, which are currently studied in order to serve as surrogate measures for RA diagnosis.

## Imaging biomarkers in arthritis: the role of ultrasound

**Figure 1:**
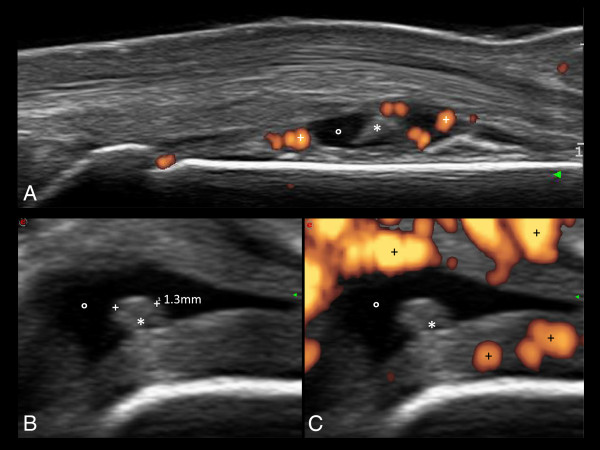
**Early arthritis.** The longitudinal dorsal scan of the II metacarpophalangeal joint **(A)** shows a wide spectrum of inflammatory findings, such as joint cavity widening, fluid collection (°), synovial hypertrophy (*) and multiple power Doppler spots (+). The transverse scans of the same joint **(B, C)** better confirm the presence of a highly perfused synovial pannus that is a strong predictor of anatomical damage. This figure is original and has not been previously published.

**Figure 2:**
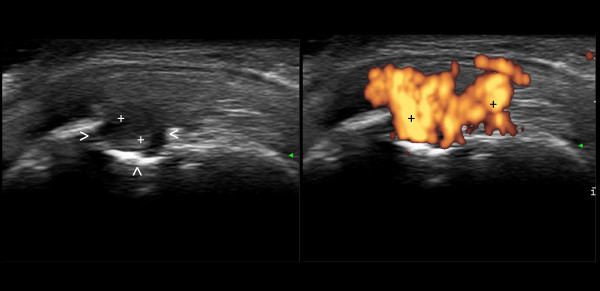
**Late arthritis (detail of the metacarpal head).** Large subchondral bone erosion (>) filled by highly perfused synovial pannus (+) that confirms the presence of intense inflammatory activity and indicates an evident unresponsiveness to treatment. This figure is original and has not been previously published.

Ultrasound (US) is able to provide high resolution multiplanar images of soft tissue, cartilage and bone profiles [4]. The high resolution of the latest generation of ultrasound equipment allows for a detailed assessment of the finest anatomical changes, which is valuable for the early diagnosis and monitoring of chronic arthritis [5]. Information obtained using US can be integrated with clinical data in patients with early disease. This leads to a more precise diagnosis based on the identification of the specific anatomical targets of the disease, especially in patients with seronegative RA [6]. It is not easy to summarize the wide range of US findings that may be candidates for the role of useful diagnostic and prognostic biomarkers in patients with arthritis [7]. These include: fluid collections, synovial hypertrophy, cartilage abnormalities, bone erosions, crystal aggregates, tendon damage, entesophytes, increased soft tissue perfusion (Figures 1 and 2).

The presence of homogeneously anechoic fluid collection without synovial hypertrophy is a reliable indicator of nonaggressive synovitis. Synovial hypertrophy is one of the most characteristic features of chronic synovitis and should be regarded as one of the most reliable morphological biomarkers of aggressive arthritis. US images of synovial hypertrophy show a significant degree of variability, from circumscribed polypoid (Figure 1B) or bushy appearance to diffuse aspects.

US allows detailed analysis of the extent and distribution of the various features of cartilage damage. In patients with advanced arthritis, cartilage damage worsens as the disease progresses, leading to progressive thinning of the joint cartilage that appears as homogeneous joint space narrowing on X-rays.

Bone erosions are the most dramatic evidence of the destructive potential of chronic arthritis. The sensitivity of US is such that bone erosions as small as one-tenth of a millimeter can be detected. Loss of sharpness and fine irregularities of the bone profile at the points of contact with the synovial pannus are probably the most sensitive morphological biomarkers to predict the subsequent appearance of erosions. The superiority of US compared with traditional radiology is due to the combination of higher spatial resolution and multiplanar exploration. The presence of synovial pannus and Doppler signal within the erosion is clinically relevant, and provides indications on the course of inflammation and the potential evolution of the anatomical damage (Figure 2).

Ultrasound is the method of choice for the examination of tendons because it provides higher spatial resolution than other imaging modalities and can be used to examine in detail the internal structure of tendons and their perfusion. The most frequent sonographic abnormalities of tendons with synovial sheath in rheumatic diseases include: tendon sheath widening, inhomogeneity of tendon structure, localized reduction of tendon diameter, contour defect, synovial cysts, interruption, fragmentation and disappearance of echotexture, tendon tear.

The enthesis is a microscopic universe that can be accurately explored with high-resolution ultrasound imaging. In gray scale imaging, the main evidence of inflammation is circumscribed or large hypo-echoic areas on the tendon part of the enthesis that can be associated with tendon thickening. Increased blood flow at the tendon insertion is generally related to the intensity of inflammation.

**Figure 3:**
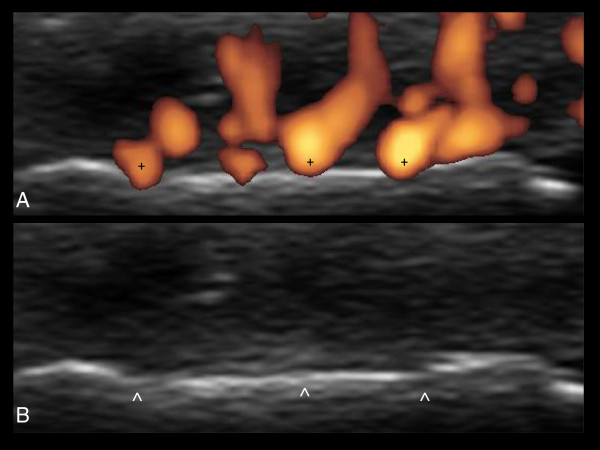
**Early aggressive arthritis (dorsal view, transverse scan).** The Doppler signal is closely linked to the bone margin **(A)**. The same image without Doppler signal **(B)** shows an evident circumscribed loss of sharpness of the bone margin (>) at the point of contact with the synovial pannus (+). This figure is original and has not been previously published.

Doppler US has proved to be a useful tool for evaluating soft tissue hyperemia [6]. Intra-articular Doppler signal in patients with chronic arthritis is mainly due to ongoing angiogenesis in areas of synovial hypertrophy. The persistence of intensely perfused areas of synovial hypertrophy inside the joint is a reliable indicator of inadequate response to therapy. Patients with this type of active synovitis should be carefully monitored and their treatment schedules modified, even if clinical response appears encouraging. The presence of synovial pannus is not limited to the joint in patients with chronic arthritis. Tendons lined by synovial sheaths may develop synovitis. Areas of synovial hypertrophy surrounding tendons may be associated with synovial fluid collection or be the unique and dominant expression of the inflammatory process. The widely ranging intensity and distribution of Doppler signal within and around a joint make establishing effective and reproducible parameters difficult. A detailed assessment of the joint cavity and of the bone and cartilage profile to identify critical areas is paramount to monitoring the disease course and the progression of anatomical damage in the short term [8]. Special attention should be paid to identifying areas where the perfused synovial pannus is in close contact with bone or cartilage. In these areas, early signs of circumscribed anatomical damage can be seen (Figure 3).

## Laboratory biomarkers in arthritis: predictive and prognostic values

Rheumatoid factor occurs in 60 to 80% of established and 50 to 60% of early RA and, until now, is one of the most widely used biomarkers in RA diagnostics [9]. RF is a relatively good biomarker for establishing the diagnosis of RA, although it does not correlate with disease activity, and is present in other systemic autoimmune as well as infectious diseases and inflammatory conditions.

Anti-RA 33, an IgG antibody directed against a nuclear protein antigen, seems to be identical to the A2 protein of the heterogeneous nuclear ribonucleoprotein (hnRNP), was detected in approximately 30% of RA and in 27 to 45% of seronegative RA patients [10]. Autoantibodies against the hnRNP A2 protein occurs in about 35% of RA patients and can be found also in other systemic autoimmune diseases, and in less than 5% of healthy individuals.

Anti-Savoie (Anti-Sa), a RA-specific autoantibody, presents in the sera of about 43% of RA patients but not in many other autoimmune diseases or in healthy individuals. In addition, 27% of RF negative RA patients were also positive for anti-Sa [11]. The overall specificity of anti-Sa is 92 to 98%, whereas the sensitivity is about 40%. The high specificity is coupled with substantial prognostic value as anti-Sa positivity has been associated with more active and destructive disease. Thus, anti-Sa might have important diagnostic and prognostic relevance in RA.

ACPAs have recently emerged as highly sensitive and specific serological markers of RA, providing a superior alternative to the RF test in the laboratory diagnostics of RA. The association of RA with shared epitope positive HLA-DRB1 alleles is stronger in ACPAs positive than in ACPAs negative RA [12]. ACPAs production can precede the onset of RA symptoms by years and ACPA-positive individuals with undifferentiated arthritis have higher risk of developing RA [13]. ACPAs have an important prognostic role, while they are associated with pronounced radiographic progression [14]. ACPAs belong to a heterogeneous family of autoantibodies, including, among others, anti-perinuclear factor (APF; targeting pro-filaggrin), anti-keratin antibodies (AKA; targeting filaggrin), and other citrullinated protein antibodies, such as citrullinated fibrinogen, histone or myelin-basic protein [15]. Anti-Sa antibodies are in fact antibodies against citrullinated vimentin and represent key autoantibodies of the ACPA family, where vimentin is secreted and citrullinated by macrophages in response to apoptosis or by pro-inflammatory cytokines [16]. Mutated isoform of vimentin gave rise to anti-mutated citrullinated vimentin (anti-MCV) ELISA [16]. The occurrence of anti-MCV is between 21 to 43% in RA, while in other systemic autoimmune diseases only 1%, which makes the autoantibody having low sensitivity, but very high specificity. APF occurs 40 to 70% of RA patients and is highly specific (80 to 90%) [17]. AKA occurs in 40 to 60% of RA patients with a rather high specificity of 80 to 95% [18]. APF and AKA can serve as early markers, since both can be detectable before clinical symptoms appear [17,18]. Diagnostic performance of antibodies to citrullinated fibrinogen (ACF) is similar to the anti-CCP2 assays. ACF is a useful tool for early diagnosis and evaluating radiographic progression of RA. Previously, the association between HLA-DRB1*0404 allele and ACF has been described [19].

ACPAs isotype distribution does not expand during disease progression from the undifferentiated arthritis to RA and is relatively stable over time. In RA, the baseline ACPAs isotype profile was a significant predictor of disease severity, with more isotypes indicating a higher risk of radiographic damage [20]. Among ACPAs, anti-CCP has a superior diagnostic and prognostic value. Table 1 summarizes the diagnostic value of various ACPA assays [21-24]. Anti-CCP and IgA-RF predict the development of RA, with anti-CCP antibody having the highest predictive value [25]. RF (IgM, IgA isotypes) and anti-CCP associated with more severe disease indicated by more erosions and severe functional impairment. The presence of anti-MCV also predicted joint damage, and the strength of this prediction was at least as strong as for anti-CCP. Higher anti-MCV levels add prognostic information compared to their mere presence or absence [26].

**Table 1:** Diagnostic performance of various anti-citrullinated protein/peptide antibodies assays in rheumatoid arthritis

	**Sensitivity (%)**	**Specificity (%)**	**Supplementary information**	**Ref.**
**CCP**	60 to 80	95 to 99	- High significant predictive value	[11,21]
			- Anti-CCP is a constant feature of RA with 5% changes in disease course	
**CCP1**	44 to 56	90 to 97	- Peptide from filaggrin protein	[22]
**CCP2**	60 to 80	96 to 98	- Artificially optimized peptide	[21,22,24]
			- Positive in 20 to 30% of RF-negative RA patients	
**CCP3**	61 to 83	93 to 98	- Artificially optimized peptide	[22-24]
			- In early and RF-neg RA patients more prevalent, with higher sensitivity/specificity then CCP2 assays	
**CCP3.1**	54 to 70	94 to 99	- FDA approved for early detection of RA	[23]
**MCV**	60 to 69	87 to 98	- Similar diagnostic performance as CCP2	[21,23,24]
			- Useful in RF-neg, anti-CCP-neg RA patients	
			- 10% of CCP-neg and 30% of IgM RF-neg RA patients are MCV positive	
			- Simultaneous CCP and MCV assessment improves RA diagnostics to ca. 98%	

CCP, cyclic citrullinated peptides; MCV, modified citrullinated vimentin; RA, rheumatoid arthritis; RF, rheumatoid factor.

## Novel serological markers

Recently, novel antibodies in RA patients have been described. For instance, anticarbamylated protein (anti-CarP) antibodies recognizing homocitrulline were detected in about 45% of RA patients and also, importantly, in up to 30% of ACPA-negative patients [27]. Homocitrulline is generated from a lysine residue following a reaction of cyanate. Importantly, in ACPA-negative patients, anti-CarP antibodies were associated with more severe radiographic progression [27]. Moreover, anti-CarP antibodies appear many years before the diagnosis of RA [28] and can predict the development of RA in arthralgia patients independent of anti-CCP antibodies [29]. Carbamylated fibrinogen or vimentin can serve as a target for anti-CarP antibodies. Overall, the sensitivity of anti-CarP is lower than ACPA; however, the simultaneous assessment of anti-CarP and ACPA can be very beneficial in identifying RA patients [27-29].

Using the cDNA phage display library, some novel autoantibodies were recently identified in early and seronegative RA patients with sensitivity ranging between 2 to 29% and specificity between 95 to 100%. These autoantibodies can be found in 44 to 67% ACPA negative RA patients [30]. The other group of novel serological markers identified by proteomic approach represents antibodies to PAD4 (peptidyl arginine deiminase 4) and BRAF (v raf murine sarcoma viral oncogene homolog B1) catalytic domain open new avenues to further pinpoint ACPA-negative RA patients [3].

## Conclusions

Despite many unanswered questions in the understanding of the mechanisms driving the immunological changes seen during the development of RA, there is evidence that systemic abnormalities defined as the presence of RA-related autoantibodies can occur several years before clinical symptoms appear. Serological biomarkers can be investigated as predictive factors in subjects that are likely to be at higher risk of developing RA, such as, for instance, first degree relatives of RA patients [30]. Simultaneous assessment of RFs along with various ACPA tests, and presumably with novel serological biomarkers, may be used in screening at the primary care level and may help to identify patients with early disease in subjects with symptoms without clinical arthritis and in those with undifferentiated arthritis or where the clinical judgment is doubtful [31]. In addition, combining positive US Doppler signal with clinical joint assessment can significantly improve certainty of diagnosis of RA in seronegative patients [6]. By revealing subclinical synovitis and radiographically undetected bone erosions, RA can be carefully explored with US especially in patients with early undifferentiated arthritis [32]. Therefore, the ACR/EULAR 2010 RA classification criteria [2] indicated that US may be used for confirmation of the clinical findings (joint involvement).

In conclusion, novel serological biomarkers along with joint ultrasound may provide additional benefit in diagnosis of RA, particularly in those with early and ACPA negative disease.

## Abbreviations

ACF: Antibodies to citrullinated fibrinogen; ACPAs: Anti-citrullinated protein/peptide antibodies; ACR: American College of Rheumatology; AKA: Anti-keratin antibodies; anti-CarP: Anti-carbamylated protein; APF: Anti-perinuclear factor; BRAF: V raf murine sarcoma viral oncogene homolog B1; CCP: cyclic citrullinated peptides; EULAR: European League Against Rheumatism; HLA: human leukocyte antigen; hnRNP: Heterogeneous nuclear ribonucleoprotein; Ig: Immunoglobulin; MCV: modified citrullinated vimentin; PAD4: Peptidyl arginine deiminase 4; RA: Rheumatoid arthritis; RFs: Rheumatoid factors; US: Ultrasound.

## Competing interests

All authors declare that they have no competing interests.

## Author contributions

All authors contributed to manuscript preparation and critical revision. All authors read and approved the final manuscript.
